# First-principles and quantum transport investigation of triclinic Mg_11_Ti_25_O_60_ as a high-voltage cathode material for magnesium-ion batteries

**DOI:** 10.1039/d6ra06938j

**Published:** 2026-10-29

**Authors:** Syeda Eman Zahra, Amara Yousaf, Muhammad Tayyab Raza, Abdul Majid

**Affiliations:** a Department of Physics, University of Gujrat Hafiz Hayat Campus Gujrat Pakistan abdulmajid40@yahoo.com ali1214@gachon.ac.kr; b Department of Physics, Gachon University 1342 Seongnam-daero, Sujeong-gu Seongnam-si Gyeonggi-do South Korea

## Abstract

Fabrication of functional intercalating cathode matrices is still a tough challenge for commercialization of rechargeable magnesium-ion batteries (MIBs). In general, the lattice stress and slow diffusion rate of Mg^2+^ ions are severe in conventional host materials. In order to tackle this multi-electron problem, a novel triclinic Mg_11_Ti_25_O_60_ cathode framework is studied comprehensively using first principles calculations based on Density Functional Based Tight Binding (DFTB) and Non-Equilibrium Green's Function (NEGF) formalisms. Our atomistic simulations indicate that the extraction of magnesium is a highly coordinated, structural mechanism, where the magnesium is removed one at a time. In general, the divalent insertion strain is well accommodated by the structurally robust Ti–O framework, and the strong local coulombic interactions result in small thermodynamic fluctuations at discrete intermediate extraction steps, ensuring the long-term structural and volume stability. Electronically, the pristine material's performance is not restricted by the resistance barriers found with traditional oxide hosts, and shows metallic behavior. The material exhibits a theoretical specific capacity of 243.25 mA h g^−1^, energy density of 819.75 W h kg^−1^ and open circuit voltage (OCV) value of 3.37 V. The results obtained from NEGF-based quantum transport calculations show good energetic compatibility between the interface and the standard ethylene carbonate electrolytes. These results, coupled with the *ab initio* molecular dynamics (AIMD) demonstrate thermal stability at operating conditions and a reasonable migration barrier of 0.62 eV. The findings of this study predict Mg_11_Ti_25_O_60_ as a promising high-voltage candidate for use in next-generation multivalent energy storage devices.

## Abstract 2

Mg_11_Ti_25_O_60_ framework is studied using DFTB and NEGF formalisms to evaluate its potential for application as cathode in MIBs.

## Graphical abstract

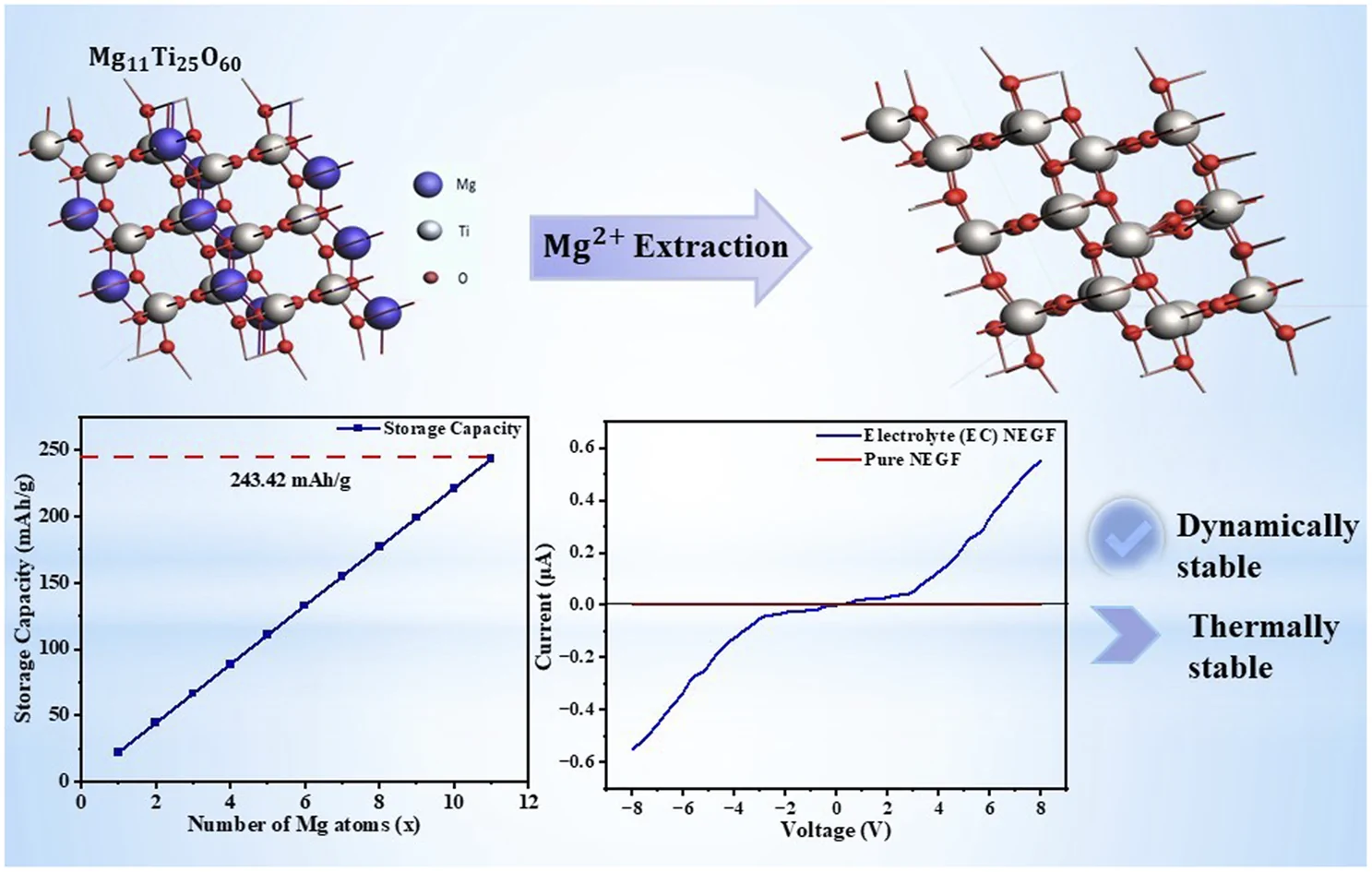

## 1. Introduction

Global applications of electrochemical technologies with a reliable energy storage capability in portable applications, in electric vehicles, and renewable energy integration at the grid-scale are rapidly increasing.^1,2^ The conventional lithium-ion battery (LIB) is currently the most successful commercial battery with promising energy density and cycling performance. However, the expanding geopolitical restrictions on lithium resources, as well as the increased production cost and critical safety issues are posing threats to its sustainability. Notably, lithium anodes have a high internal surface energy, which can lead to the uncontrolled growth of crystalline dendrites during fast charge and discharge cycles and cause internal short circuit, mechanical penetration of the separator, and thermal runaway.^3^ This has led to the development of rechargeable magnesium ion batteries (MIBs) as a very attractive alternative battery technology for next generation energy storage.^4^ Magnesium is highly abundant (1.35 wt% of the earth's crust) and it can be extracted from natural mineral deposits and seawater in an environmentally friendly manner.^5^ Magnesium anodes have a competitive reduction potential of −2.37 V *versus* the standard hydrogen electrode (SHE), and an excellent gravimetric capacity of 2205 mA h g^−1^ and a volumetric capacity of 3883 mA h cm^−3^.^6–8^ Moreover, Mg^2+^ is a divalent ion, so it can undergo a two-electron transfer for each ion, directly improving the volumetric energy density. Most importantly, the divalent electrodeposition mechanism proceeds in a very uniform, non-dendritic morphology, and completely removes the risk of short circuit and explosive cell degradation even under the most aggressive high-current conditions. These exceptional properties make MIBs particularly ideal for stationary power systems, electric transportation and significant recent advances have been made also on the long-standing problems of the magnesium metal anode and the electrolyte chemistry. In recent years, improving charge transfer at the interface between the electrode and electrolyte has been achieved by controlling electric fields at the interface, which appeared to improve both the Mg plating kinetics and the cycling stability.^9^ Further, significant work has been reported on formation of alternative electrolytes that are not corrosive to both the electrolyte and components.^10^ The electrolytes exhibit high stability across the electrochemical window, with the exception of chloride-based electrolytes. Additionally, the utilization of weakly coordinating co-solvent approaches has been shown to control the solvation structure of the Mg-ions and their interfacial chemistry, promoting the reversible deposition of Mg and electrochemical reversibility.^11^ The importance of the formation and regulation process of the anode/electrolyte interphase (AEI) in controlling the Mg ion transport, electron transfer and electrochemical stability of rechargeable magnesium batteries has also been highlighted in the recent literature.^12^ Therefore, rational design of cathode materials which can possess strong frameworks, open diffusion paths, and promising Mg-ion storage properties is a key challenge to the realization of high-performance MIBs.

Although significant efforts have been made in MIBs research, there is still a significant hurdle to commercializing the batteries, namely the necessity of functional intercalating cathode host matrices. The root of the primary obstacle is the high charge density of Mg^2+^, which has strong coulombic attractions to neighboring anions in host lattices. This close association results in slow solid-state diffusion kinetics, high migration activation energy, poor ionic conductivity, and poor rate capability.^13–15^ In addition to these, conventional MIB cathodes have operating voltage limitations and a limited capacity. Mo_6_S_8_, is a classic Chevrel phase material which is reversible with good capacity (100–130 mA h g^−1^) but operates within a relatively low voltage of 1.2–1.4 V, limiting the practical energy density below 300 W h kg^−1^. The high-capacity vanadium-based materials, such as layered V_2_O_5_ has extreme degradation, poor long-term cycling stability, and severe voltage drops (2.3–2.5 V). Conversely, two-layer transition metal sulfides such as TiO_2_ and MoS_2_ exhibit promising interlayer spacing; however, capacity fading is often severe. Low values of the diffusion coefficient promote sluggish ion mobility.^16–19^

Of all the candidates explored to address these multi-electron challenges, titanium-based oxides have been studied extensively because they are structurally robust, chemically stable, environmentally friendly, and can host multivalent ions. The redox centers of the Ti ions (Ti^3+^, Ti^4+^) allow reversible electrochemical reactions, and the rigid oxide structure ensures preservation of the structure throughout several magnesiation and demagnesiation cycles.^20–23^ While traditionally used pristine titanium oxide, such as anatase TiO_2,_ often presents low electronic basal conductivities as well as localized kinetic trapping phenomena, proper structural design and stoichiometric optimization are necessary to achieve open ion channels. Considering these synergic benefits, the present study comprehensively investigates and evaluates the novel Mg_11_Ti_25_O_60_ as a cathode material for rechargeable MIBs. We systematically studied structural properties, electronic band gap behaviors, charge-transfer dynamics, thermal properties, OCV, storage capacity, ionic conductivity and energy density of the system, using first-principles findings assisted by Density Functional based Tight Binding (DFTB) and Non-Equilibrium Green's Function (NEGF) formalisms. Moreover, the synergetic interaction between the Mg, Ti and O atoms facilitates and enhances the reversible ion storage of Mg^2+^ ions and the electrochemical capabilities of the material. To the best of our knowledge, Mg_11_Ti_25_O_60_ has not been previously reported as a cathode material for use in rechargeable magnesium-ion batteries. The findings of this first-principles study demonstrate that Mg_11_Ti_25_O_60_ is a promising high-voltage cathode candidate for multivalent energy-storage applications and furnish a theoretical basis for future experimental follow up.

## 2. Computational details

The structural stability and electrochemical activity of triclinic Mg_11_Ti_25_O_60_ as a possible candidate cathode material for MIB have been calculated from first principles. Density functional based tight-binding (DFTB) module of the Amsterdam Modeling Suite (AMS/ADF) software package was used for all structural simulations, using a 96 atom unit cell. The multivalent framework was relatively large, but to ensure a high computational accuracy, the semi-empirical GFN1-xTB method was used efficiently.^24^ The host structure was first relaxed as much as possible to obtain the lowest energy configuration of the structure, with unrestricted relaxation of all atomic coordinates, *via* the geometry optimization (GO), and then rigidly relaxed with only the geometry of the atoms of the host structure optimized, but the atomic coordinates of the atoms of the guest structure were kept fixed. The intermediate intercalation states of Mg_11_Ti_25_O_60_, and the isolated constituent atoms (Mg, Ti, and O) were optimized similarly. The thermodynamic and structural stability of the framework were thoroughly investigated after the optimization, based on the calculation of its formation energy (*E*_f_) using the standard formalism:^25,26^

1 *E*_f_ = *E*_defect_ − *E*_pristine_ − ∑*n*_i_*µ*_i_

in eqn (1), *µ*_i_ is defined as the chemical potential and *n*_i_ is the number of atoms adsorbed/removed. The value of extraction energy (*E*_ext_) was calculated for the entire charge–discharge process. The *E*_ext_ is the energy needed to detach a Mg atom from the crystal. The lower extraction energies suggest that Mg can easily be removed and *vice versa*, which is calculated by eqn (2).^27,28^2
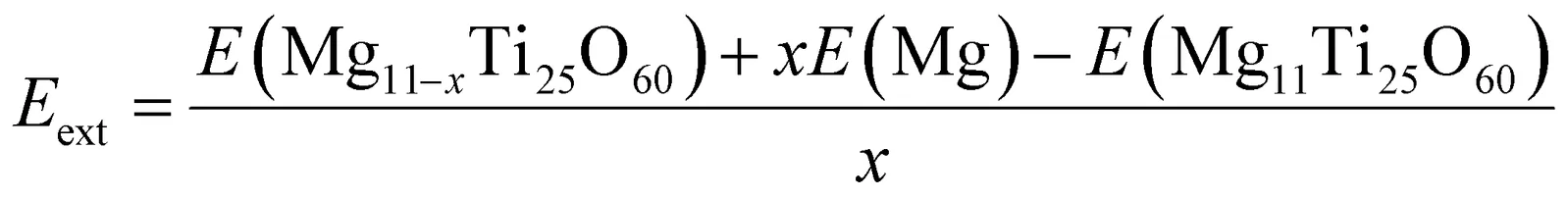
*E*(Mg_11−*x*_Ti_25_O_60_) is the energy after removal of Mg, *x* defines the number of Mg removed, *E*(Mg_11_Ti_25_O_60_) is pristine structure's energy and *E*(Mg) is the energy of Mg atom (isolated).

Single-point (SP) calculations were performed with the BAND module, considering the generalization of the exchange–correlation functional (GGA) within the Perdew–Burke–Ernzerhof (PBE) scheme, and triple-zeta polarization (TZP) basis sets. It is used to give insights to the cathode material's electronic behavior by band structure and density of states (DOS), in particular, which can show the band gap and the contribution of the atomic orbitals.^29–33^

Phonon dispersion calculations were executed to investigate the dynamic stability of Mg_11_Ti_25_O_60_. Vibrations of the crystal lattice and dynamics can be investigated using phonon analysis.^34,35^ The *ab initio* molecular dynamics (AIMD) simulations were carried to evaluate thermal stability of the pristine and fully demagnesiated structures using the canonical NVT ensemble and Nosé–Hoover thermostat. The total simulation time was 500 ps with a time step of 1.0 fs for each simulation to gauge the finite temperature structural robustness of the material.^36,37^ The interfacial charge transfer and electronic redistribution have been investigated by Hirshfeld charge analysis.^38^

The practical application performance of the cathode framework was evaluated by deriving crucial macroscopic metrics for the battery, such as theoretical specific storage capacity, open-circuit voltage (OCV), and energy density from the first-principles data. Specific capacity is the amount of charge a battery can hold reversibly per gram of cathode material and is an important parameter to characterize its performance. It can be determined by using eqn (3).^39^3
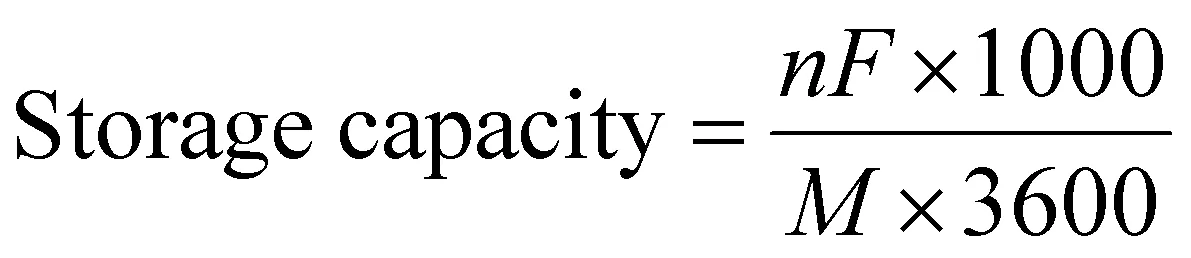
*n* indicates number of particles involved in redox reaction, *F* is equal to 96 485 C mol^−1^ called the Faraday constant and *M* = 2423.97 g mol^−1^ known as molar mass of Mg_11_Ti_25_O_60_. The electrochemical potential (OCV) supplies information about battery performance as well as energy density and can be calculated using eqn (4).^39–41^4
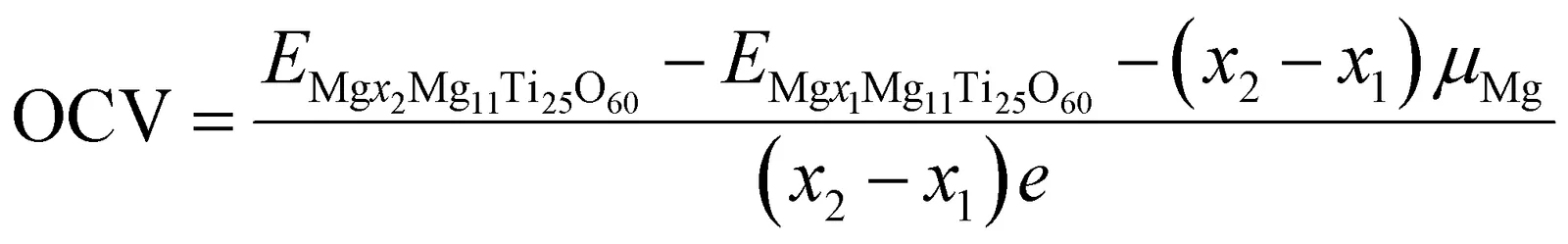


Energy density is an important characteristic of battery systems used in high-energy storage systems, indicating the energy delivered per mass of the cathode material. It is calculated by eqn (5).^42^

5 Energy density = storage capacity × *V*_avg_

The energy barriers were modeled using the Climbing Image Nudged Elastic Band (CI-NEB) method. A set of intermediate images was relaxed between the stable initial and final insertion positions to correctly approximate the Minimum Energy Path (MEP). The forces operating on these images is defined per eqn (6).^43–46^

6 *F*_i_ = |*F*^s^|_∥_ − |∇*E*(*R*_i_)|_⊥_

*F*_i_ demonstrate the net force acting on the *i*-th image, the tangential component of the elastic force is ∣*F*^s^∣_∥_ and the normal component of the real potential force is ∣∇*E*(*R*_i_)∣_⊥_. The expression (7) is used to approach the transition state to find the exact saddle of this point of the maximum energy image:^47^

7 *R*_i_max = ∇*E*(*R*_i_max) + 2∇*E*|(*R*_i_max)|_∥_

Furthermore, per eqn (8) and (9), the calculations of mean square displacement (MSD) were performed *via* MD at 300 K to calculate the diffusion co-efficient (DC), and ionic conductivity (*σ*) in accordance with the Nernst–Einstein relation.^48^8
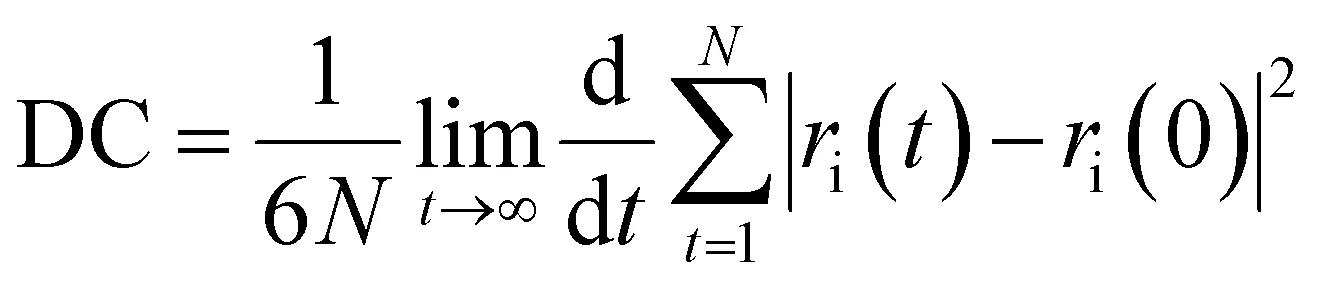

9 MSD = *r*(*t*) − *r*(0)^2^

in the above equations, *r*(*t*) and *r*(0) are position vectors. The ionic conductivity was calculated using eqn (10):^49–51^10
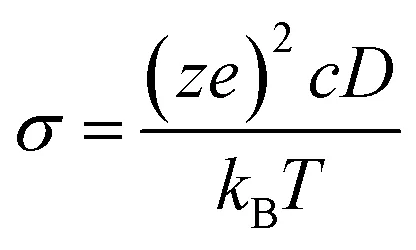
*z* is ionic valence, *e* is charge, and *c* is the ion concentration. Thus, the Boltzmann constant is indicated by *k*_B_, where *T* indicates the temperature, and *D* indicates the diffusion parameter. The current–voltage (*I*–*V*) properties were governed by a Non-Equilibrium Green's Function (NEGF) formalism. Under external bias conditions, behavior of electrons across interface can be assessed *via* the transport analysis per Landauer–Büttiker formulism.^52,53^

## 3. Results and discussion

A comprehensive theoretical study was carried out to assess Mg_11_Ti_25_O_60_ as a cathode material for MIBs. Systematic characterization of its structural, electronic, diffusion, and electrochemical properties was carried out to gain an understanding of Mg storage mechanism and evaluate its capability for next-generation energy storage applications. The findings of the work are described in the following sections.

### 3.1 Structural properties

The proposed novel cathode material, Mg_11_Ti_25_O_60_ which is predicted to crystallize in a triclinic system of *P*1 space group, is shown in Fig. 1. It demonstrates a symmetry reduction relative to the parent monoclinic system Mg_5_Ti_13_O_30_ phase due to increased Mg stoichiometry. The optimized lattice parameters are calculated as *a* = 10.51 Å, *b* = 10.58 Å, and *c* = 10.10 Å, with inter-axial angles of *α* = 99.54°, *β* = 110.01°, and *γ* = 111.07°. The total unit cell volume is determined to be 1124.20 Å^3^, yielding a volume per atom of 11.70 Å^3^. This value is consistent with titanium-based oxide frameworks, such as anatase TiO_2_ that are known to be viable as hosts of Mg^2+^.^54^ When compared to the Mg_5_Ti_13_O_30_, which is 11.42 Å^3^ of monoclinic symmetry (mp-759796), a slight lattice expansion is observed. This growth is taken physically when they accommodate more ions of Mg^2+^ in the titanium oxide structure. The bond lengths for the host material Mg_11_Ti_25_O_60_ have been defined on average between certain atoms like Ti–O and Mg–O as 2.05 Å and 2.03 Å respectively. Formation energy (*E*_f_) for the optimized structure is −2.27 eV, as examined by the experimental synthesis of thermal properties with the relevant literature.^26,55–59^

**Fig. 1:**
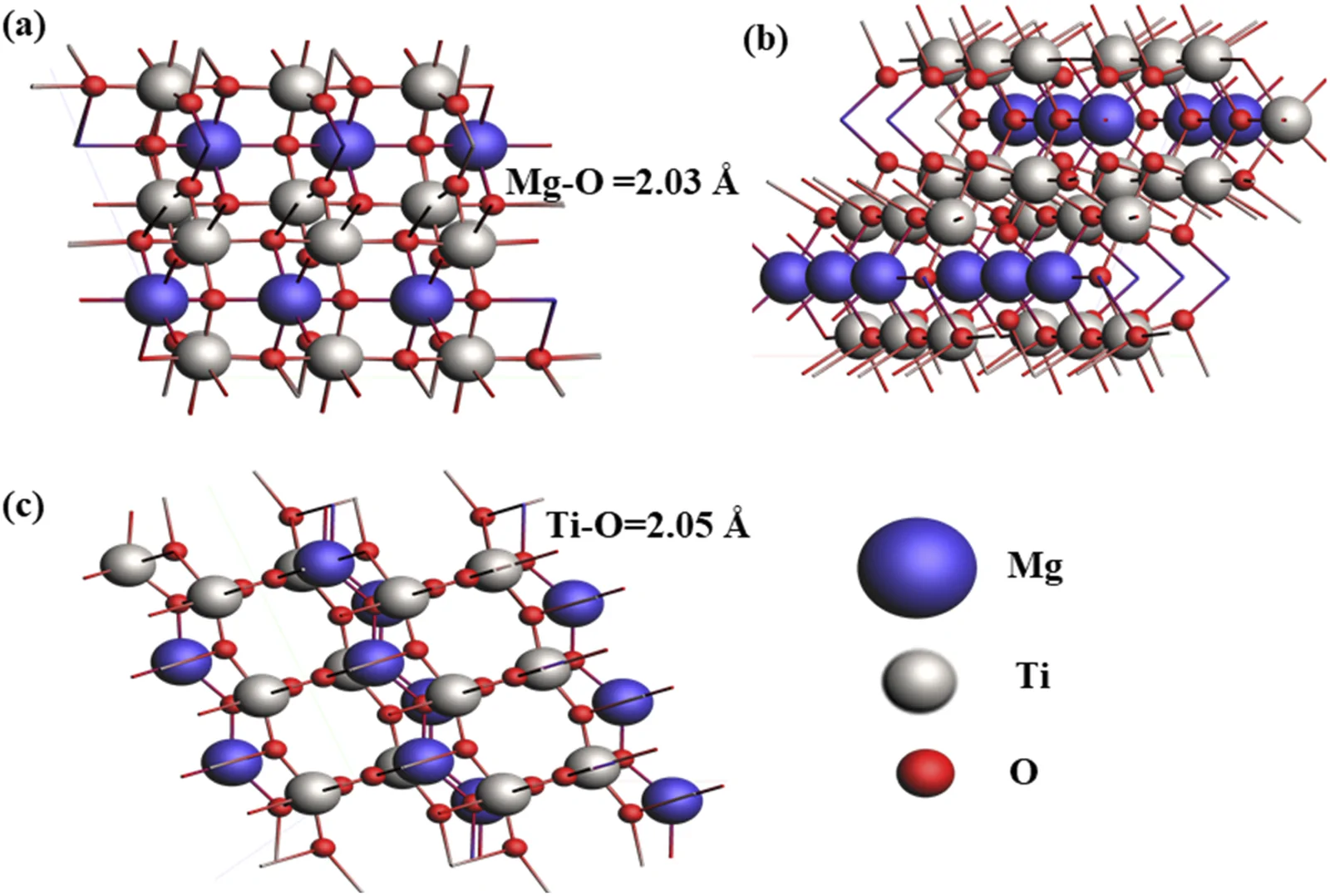
The optimized structure of Mg_11_Ti_25_O_60_ viewed along (a) *x*-axis, (b) *y*-axis and (c) *z*-axis.

### 3.2 Demagnesiation energetics

The electrochemical de-intercalation performance of the proposed cathode material, Mg_11_Ti_25_O_60_ was evaluated through a systematic demagnesiation study with sequential removal of Mg atoms from a fully optimized host material. The mechanism of the extraction of Mg^2+^ from the framework is given in (11):

11 Mg_11_Ti_25_O_60_ → Mg_11−*x*_Ti_25_O_60_ + *x*Mg^2+^ + 2*x*e^−^

where *x* is the concentration of Mg^2+^ extracted and it ranges between 0 and 11; for complete demagnesiation, shown in Fig. 2. The energies calculated for the extraction are calculated from eqn (2), shown in Table 1. *E*_ext_ rises with increasing numbers of extracted Mg^2+^, reaching a maximum value of 6.92 eV for Mg_8_Ti_25_O_60_, it diminishes a little for further removal of Mg, ranging from 6.67–6.89 eV, before decreasing to 6.72 eV for a completely demagnesiated structure, graphically shown in Fig. 7(b). This behavior is related to the increase of the electrostatic interaction between the remaining Mg^2+^ ions and the Ti–O host framework. As the Mg content gets lower, the local space of the residual cations gets more constrained, and hence, more energy must be invested to remove them. It is typical behavior in multivalent cathode materials that have a vacancy concentration increasing with increasing local electrostatic potential stability of intermediate compositions.^60–62^

**Fig. 2:**
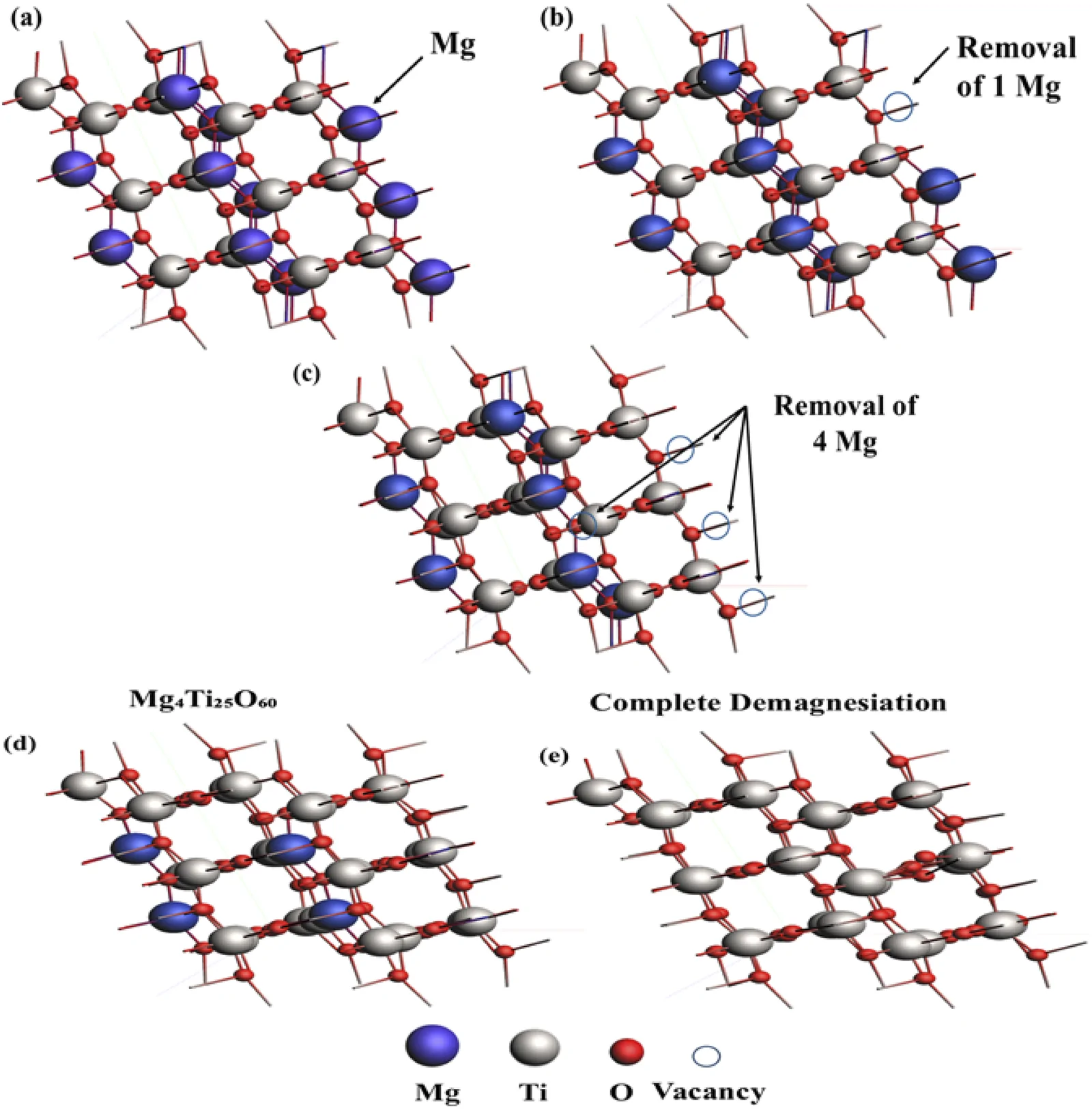
Visualization of (a) Mg_11_Ti_25_O_60_, (b) Mg_10_Ti_25_O_60_, (c) 4 Mg removed, (d) Mg_4_Ti_25_O_60_ and (e) Mg_0_Ti_25_O_60_ (fully demagnesiated).

**Table 1:** Calculated *E*_ext_, storage capacity, and OCV for a fully magnesiated to de-magnesiated structure

Configuration	*x*	*E*_ext_	Storage capacity	OCV
Mg_10_Ti_25_O_60_	1	4.56	22.11	2.33
Mg_9_Ti_25_O_60_	2	6.88	44.22	4.54
Mg_8_Ti_25_O_60_	3	6.92	66.33	3.58
Mg_7_Ti_25_O_60_	4	6.47	88.45	2.77
Mg_6_Ti_25_O_60_	5	6.54	110.56	3.28
Mg_5_Ti_25_O_60_	6	6.58	132.67	3.33
Mg_4_Ti_25_O_60_	7	6.65	154.80	3.51
Mg_3_Ti_25_O_60_	8	6.89	176.91	4.29
Mg_2_Ti_25_O_60_	9	6.85	199.02	3.27
Mg_1_Ti_25_O_60_	10	6.83	221.14	3.32
Mg_0_Ti_25_O_60_	11	6.72	243.42	2.81

Overall, *E*_ext_ calculated throughout the entire process of demagnesiation are positive, as they should be for a thermodynamically stable compound.^27,28,63^ Reversible Mg insertion/extraction needed for rechargeable Mg-ion batteries is highly desirable, which includes successful maintenance of stability across several intermediate compositions that lead to charge insertion. A sequential extraction of magnesium ions provides proof that the Mg^2+^ are the electrochemically active species of the Mg_11_Ti_25_O_60_, while the titanium–oxygen network is the main structural backbone. The Ti–O framework is well retained during demagnesiation and the structure is highly robust. Relative volume change (Δ*V*/*V*) was also used to further evaluate the structural stability of the material during Mg extraction. The comparative analysis indicates that the cell volume is reduced from 1124.20 Å^3^ to 1081.90 Å^3^ demonstrating the reduction of −3.76% (Fig. 7(c)). This small lattice strain of less than 4% indicates the excellent structural robustness of Mg_11_Ti_25_O_60_ framework and Mg removability in a reversible manner without significant lattice deformations.^64^ This magnesium vacancy generation may create extra channels for the diffusion of Mg^2+^ ions, which can enable ion mobility during cycling operation.^65^ Moreover, the extraction of magnesium is expected to affect the oxidation state of the titanium species, with progressive oxidation of Ti^3+^ species to Ti^4+^. Based on these observations, Mg_11_Ti_25_O_60_ is prospective cathode material for rechargeable MIBs, wherein reversible electrochemical activity depends mainly on such transition-metal redox activities.^66^

### 3.3 Electronic properties

The electronic response of cathode materials is important for understanding their electrochemical activity, charge-transfer behavior, and electronic conductivity.^67^ Density of states (DOS) analysis offers useful information about the nature of the orbital interactions, the distribution of charge, and the individual atomic species contribution in the region of Fermi level (*E*_F_). Moreover, band-gap engineering *via* compositional tuning has been extensively used to enhance the conductivity of transition-metal oxide cathodes in multivalent-ion batteries.^68–70^ The calculated Total Density of States (TDOS), Spin DOS and Partial Density of States (PDOS) are shown in Fig. 3. The electronic features of the materials Mg, Ti and O are given as 1s^2^2s^2^2p^6^3s^2^, [Ar] 3d^2^4s^2^ and 1s^2^2s^2^2p^4^, respectively. The analysis of the calculated TDOS indicates that electronic states are crossing the *E*_F_ shown as the vertical red dashed line, while the spin-resolved DOS (spin-up and spin-down) shows finite contributions from both channels near *E*_F_. It points to an intrinsic metallic electronic behavior of the host structure Mg_11_Ti_25_O_60_. The estimated band gap is zero and is much lower than that of pure anatase TiO_2_ (∼3.2 eV). Such behavior is very desirable to advance multivalent cathode frameworks, since the system evades the common high electronic resistance barriers present in the standard olivine or layered oxide compounds.^39,71–79^

**Fig. 3:**
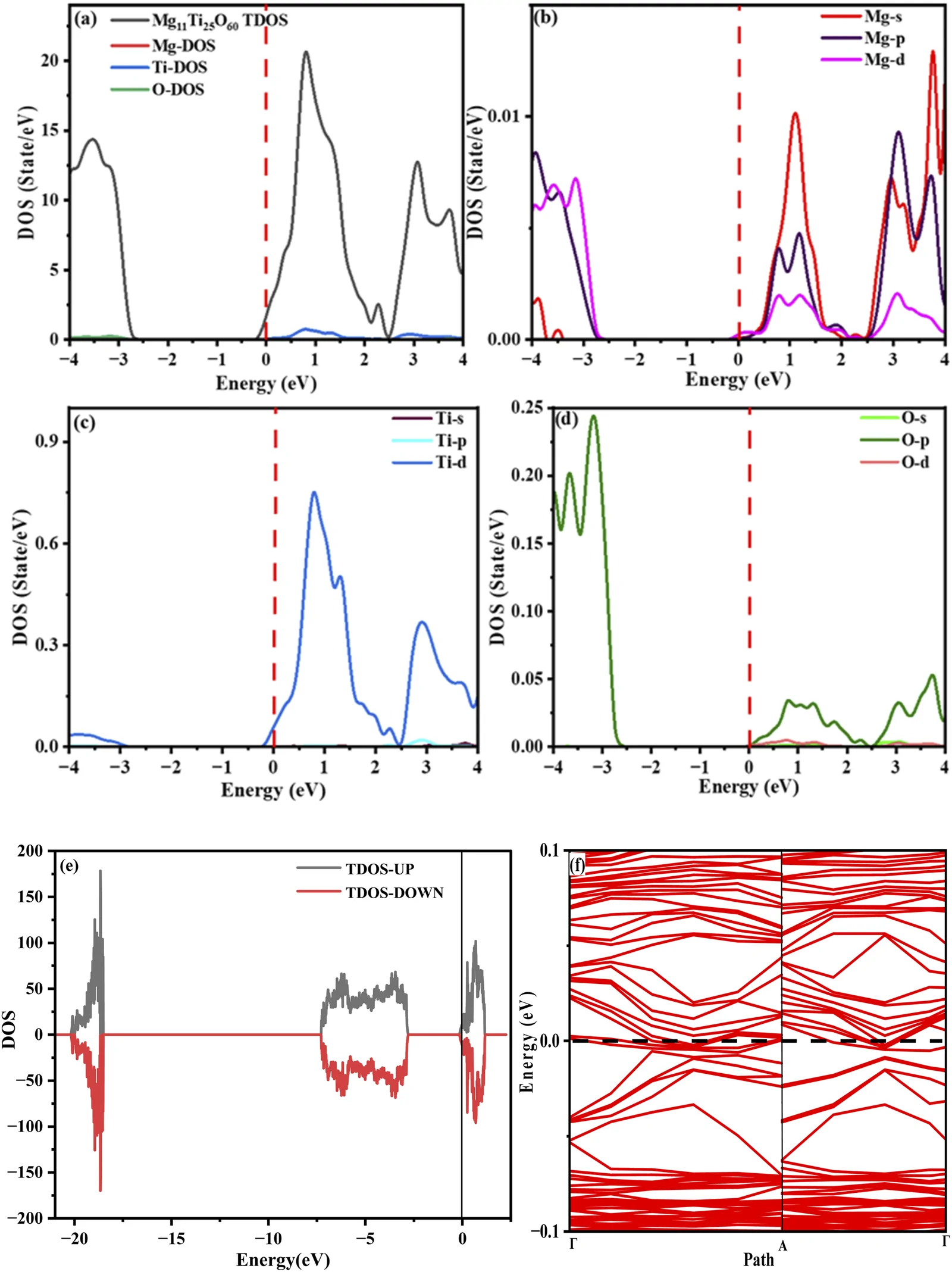
(a) TDOS of pristine Mg_11_Ti_25_O_60_, PDOS of (b) Mg, (c) Ti, (d) O atom and (e) spin DOS (f) band structure of Mg_11_Ti_25_O_60_.

The PDOS analysis shows that the electronic states near the valence band are mainly of O 2p and the electronic states near the conduction band are mainly of Ti 3d. There is significant overlap between O 2p and Ti 3d orbitals, .indicating strong O 2p/Ti 3d orbital interaction within the Ti–O framework, ensuring the electronic stability and the ionic conductivity of the material. Titanium-based oxide cathodes have also been reported to have similar electronic properties to those used in multivalent-ion batteries.^78–80^ In contrast, Mg^2+^ states (Fig. 3(b)) exhibit minimal contribution at the valence band, corresponding to sp^3^ spatial distribution. This is a clear sign that the interaction between the active Mg^2+^ species and the surrounding highly mobile oxygen coordination cages are essentially ionic, and the density of covalent bonds does not shift away from the backbone of the framework. Similar cases where the electronic gap is dramatically lowered have been extensively reported in next-generation MIB hosts, such as spinel-type Mg_2_V_2_O_5_, intermediate titanium-based configuration, yields the same bandgap closures, and retains high electronic carrier concentrations for the maintenance of state-of-the-art electrochemical functionality.^81–83^

### 3.4 Lattice dynamics and phonon dispersion analytics

Dynamic stability is an important prerequisite for the use of cathode materials in practical magnesium-ion rechargeable batteries. Phonon dispersion calculations offer first-hand knowledge of lattice stability by visualizing the vibrational behavior of the atoms in the entire Brillouin volume. If there are no imaginary (negative) phonon frequencies, this means that the crystal lattice is dynamically stable since it will not yield distortions under small atomic perturbations.^84–88^

The phonon dispersion spectra of Mg_11_Ti_25_O_60_ is displayed in Fig. 4. The phonon frequencies in the whole Brillouin zone are positive, with no negative (imaginary) modes observed, suggesting dynamical stability of the optimized structure. All optical modes are positive, and the acoustic ones start at the *Γ*-point at zero frequency. From a physical perspective, this positive energetic profile results from the interconnected TiO_6_ octahedral networks having a high restoring force that can accept lattice strain. As a result, the material is structurally stable and is not susceptible to phase destabilisation during repeated divalent ion insertion and extraction of Mg^2+^ thus providing long-term cyclic stability. The phonon stability of Mg_11_Ti_25_O_60_ is similar to other reported Mg-ion battery cathodes, such as Chevrel-phase Mg_2_Mo_6_S_8_ and Se-doped MgMo_6_S_8_, they do not have imaginary phonon frequencies throughout the Brillouin zone. The same dynamically stable phonon properties were observed also for Mg-intercalated vanadium oxide systems, and this behavior is a good indicator of structural stability for materials composed of multiple valanced ions as cathodes in rechargeable batteries.^89–91^

**Fig. 4:**
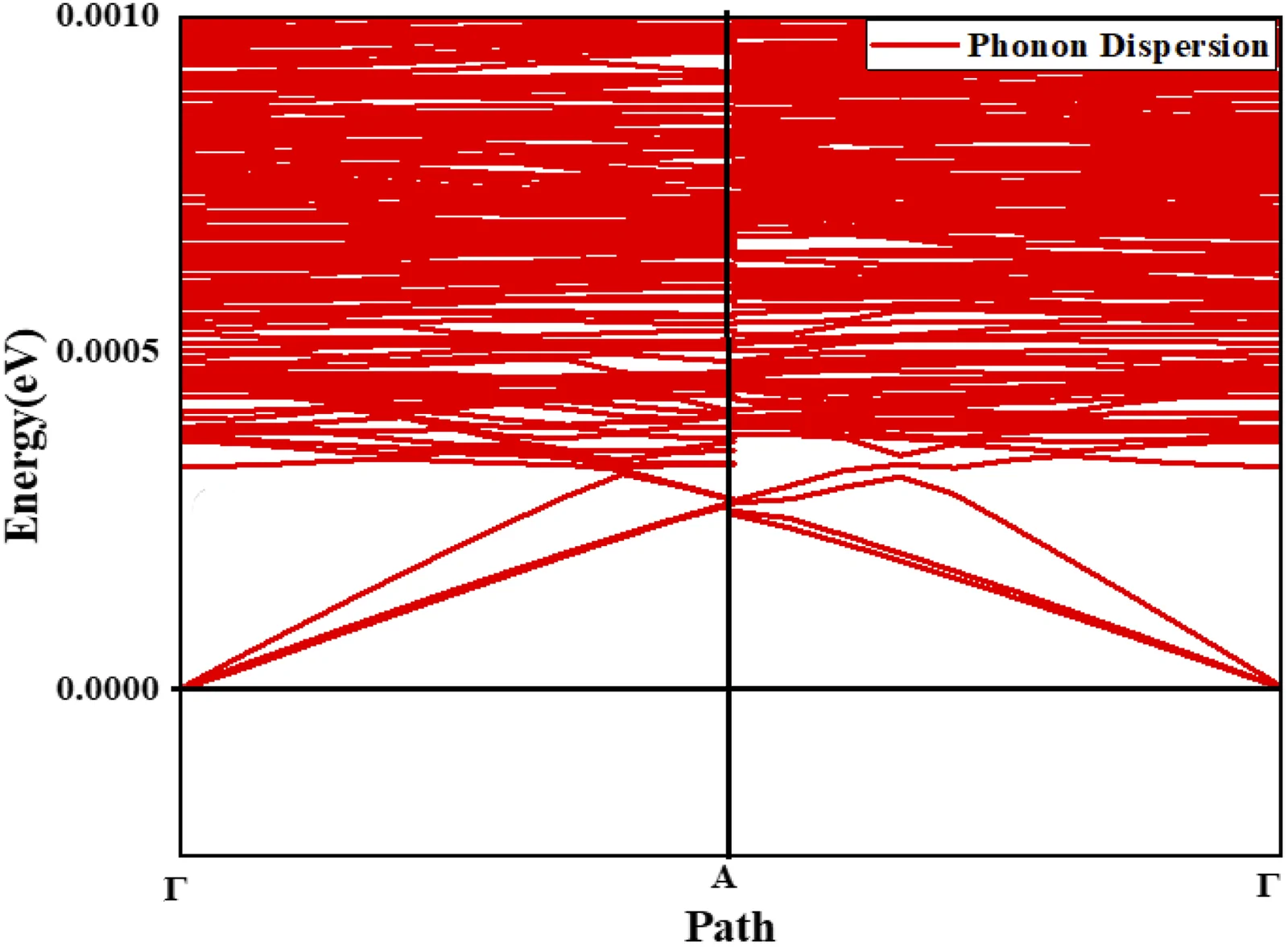
The phonon band structure dispersion spectrum of Mg_11_Ti_25_O_60_.

### 3.5 Thermal constraints and *ab initio* molecular dynamics

Thermal stability is one of the most important properties of cathode materials since batteries undergo temperature changes during cycling charges. AIMD simulations have been widely used to assess the structural robustness and thermochemical stability of the electrode materials under operation.^92–96^ The simulations for pristine Mg_11_Ti_25_O_60_ and fully demagnesiated Ti_25_O_60_ are shown in Fig. 5. In the case of Mg_11_Ti_25_O_60_ and fully demagnesiated structure, the energy becomes stabilized around 315 eV, while the fully demagnesiated structure is stable at an energy of 128 eV. There is no sudden energy change or drift in energy level throughout the simulation.^97^ These results indicate the structural integrity of both pristine and demagnesiated structures in thermal conditions. The temperature and energy evolution along the entire stable trajectory of Ti–O reveals no lattice collapse, phase changes, or dissociation of the framework even after complete Mg removal. In general, the results of the AIMD indicate that Mg_11_Ti_25_O_60_ is thermally resistant and a good candidate as a cathode material of MIBs. It is worth mentioning that the main aim of conducting AIMD simulations was to assess the thermal stability of the material. Due to relatively small simulation time of 500 ps, the findings of the AIMD simulation cannot be directly used to quantify the long-range migration of the Mg-ions. Hence, the results on Mg migration will be discussed mostly on the basis of CI-NEB calculations.

**Fig. 5:**
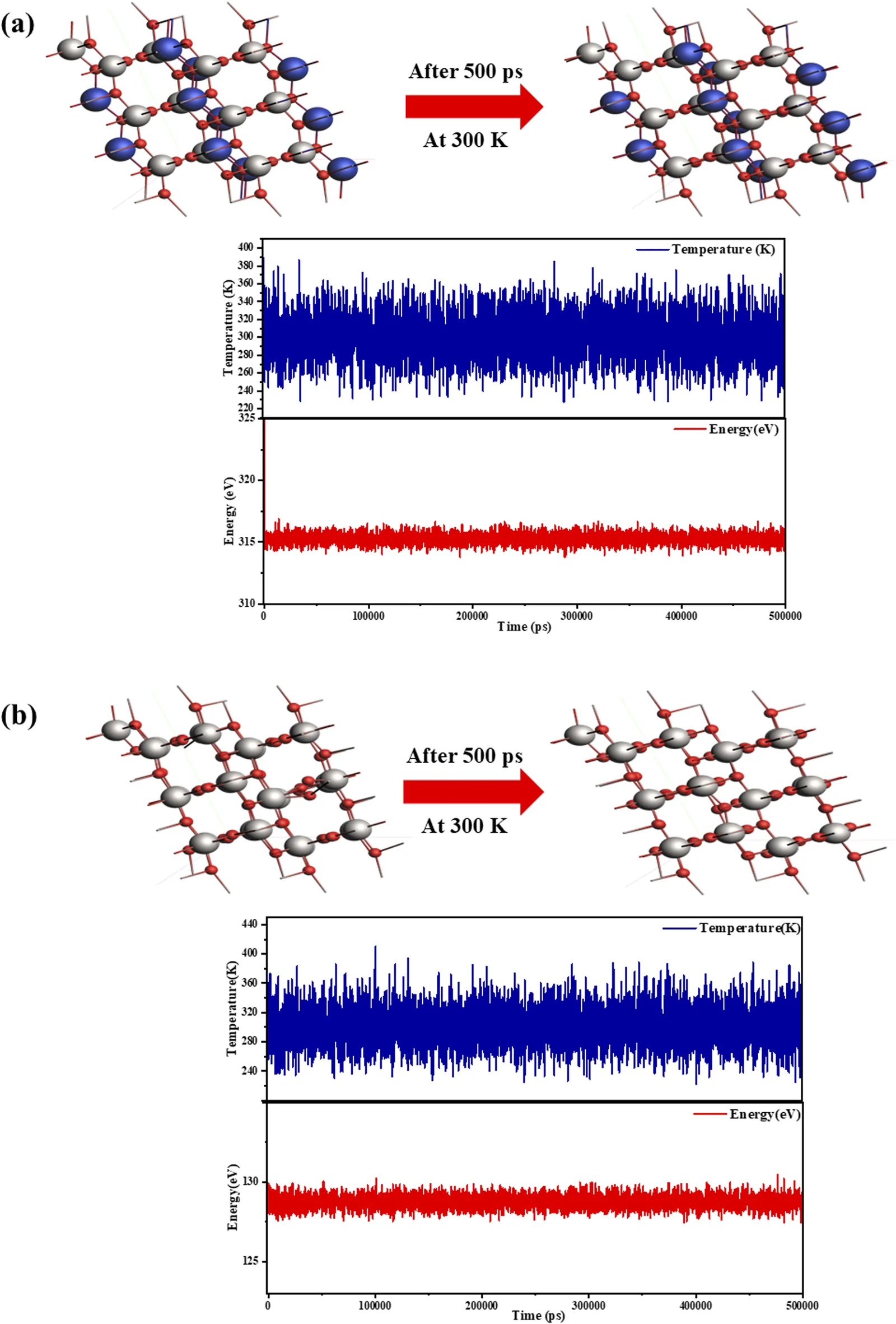
Snapshot of AIMD simulation at 300 K (a) Mg_11_Ti_25_O_60_ (b) Mg_0_Ti_25_O_60_.

### 3.6 Interfacial Hirshfeld charge redistribution mechanics

To investigate charge transfer mechanisms and localized electronic interactions in host frameworks, Hirshfeld charge analysis is performed. DOS plots capture the overall electronic picture, but Hirshfeld analysis provides a precise measure of the electronic charge transferred from atom to atom. A deep understanding of these subtle charge redistributions on the atomic scale is an essential basis for the design of stable electrode materials and for modifying the rate properties of the materials.^98–101^ The Hirshfeld charge distribution of Mg_11_Ti_25_O_60_ in the different demagnesiation processes is displayed in Fig. 6(a)–(d). For pure structure (Fig. 6(a)), there are positive charges at Mg atoms, while the negative charges at the O atoms, resulting in electron transfer from Mg to Ti–O framework. In the fully intercalated state, the atoms that are dominantly charged are Mg and Ti (1.29*e* for Mg and 1.77*e* for Ti), while oxygen atoms are negatively charged (O down to −0.783*e*). The distribution clearly shows the strong ionic character of the Mg–O bonds, which is expected in the terminal fully extracted state as shown in Fig. 6(d), where even if all the opposite ions have been removed, the stability of the framework is still high, with localized oxygen charge of −0.660*e*. The positive values of charge indicate electron donation and *vice versa*.^102^

**Fig. 6:**
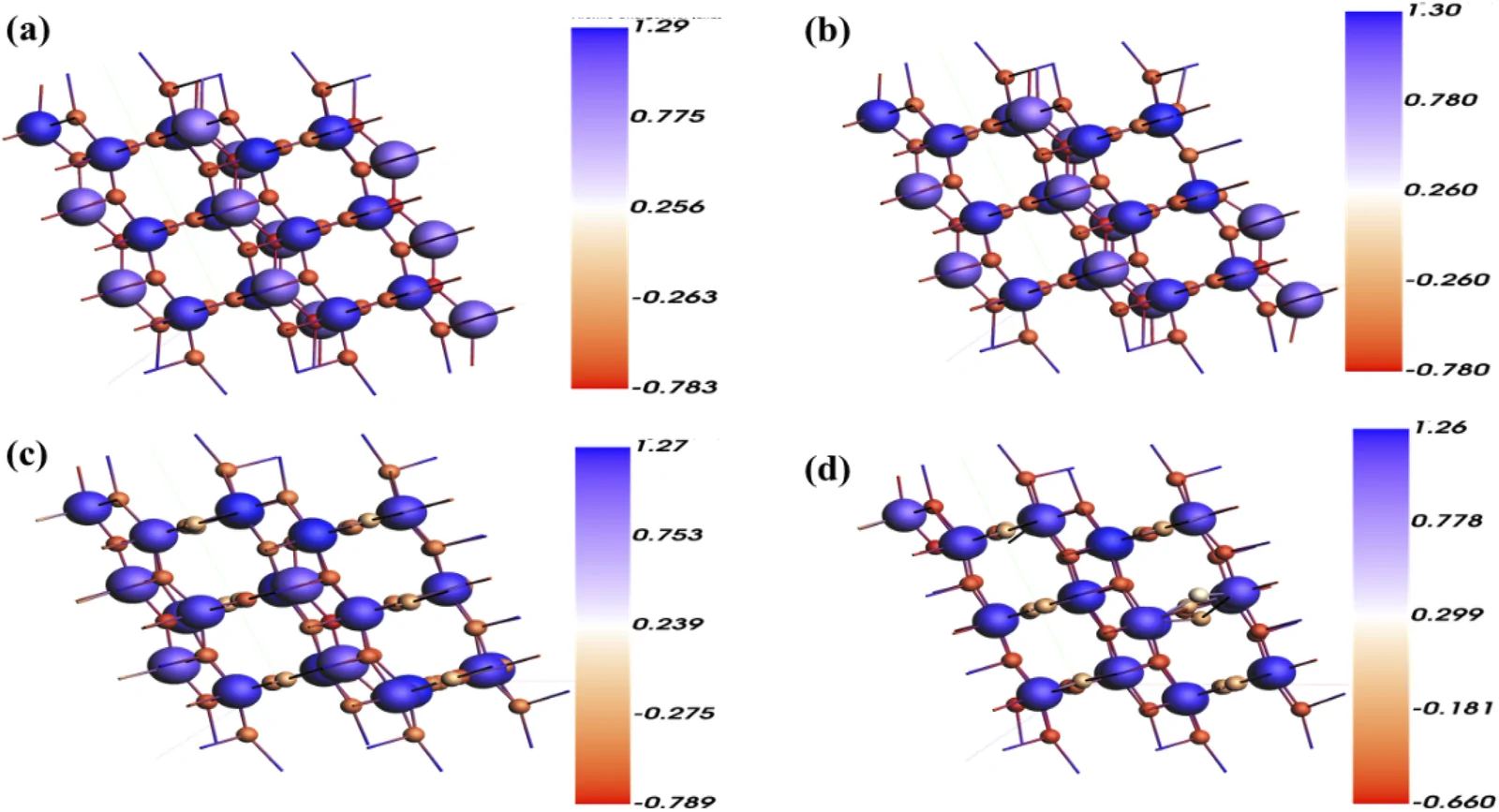
Hirshfeld analysis of Mg_11_Ti_25_O_60_ (a) pristine (b) Mg_9_Ti_25_O_60_ (c) Mg_4_Ti_25_O_60_ (d) Mg_0_Ti_25_O_60_.

The degree of demagnesiation increases, and the electronic charge is evenly distributed throughout the framework without a large charge localization. Under total demagnesiation (Fig. 6(d)), the Ti–O host structure easily accepts the electronic rearrangement, by charge compensation, and shows also quite high electronic and structural robustness. These findings indicate the Ti–O framework to have a stable charge-storage backbone and sustaining reversible insertion/extraction of Mg^2+^. This analysis confirms explicitly how the empty 3d orbitals of Ti help in the adsorption and desorption process during cycling. This flexibility of structure allows for a dynamic redistribution of charges and polarization throughout the system, and in this way the Mg_11_Ti_25_O_60_ host achieves success. This material guarantees high activation pathways for electron transfer, preventing lattice collapse and charge trapping at extreme conditions that are essential for high specific capacity, fast rate capability and long-term cycling stability in Mg-ion batteries.

### 3.7 Bulk electrochemical performance metrics

Specific capacity is one of the most crucial electrochemical parameters that is used to assess energy storage capacity of the cathodes. The high electrochemical efficiency and high-rate capability are directly related to the achieved specific capacity, which are the major pre-requisites for heavy-duty, long-cycle, and high-energy-density applications.^103–105^ The specific capacity of Mg_11_Ti_25_O_60_ was theoretically calculated with the help of Faraday's law, which is 243.25 mA h g^−1^, shown in Fig. 7(b), and written in Table 1. These values are significantly greater than several conventional MIBs cathodes, such as Chevrel-phase MgMo_6_S_8_, TiS_2_, Mo_6_S_8_ and other reported materials.^16,18,22,26,41,55,106–122^ The Prussian blue and other organic cathodes contain high storage capacity but offer limitations regarding energy density and electrochemical potential due to the restricted movement of Mg. The relatively high capacity of Mg_11_Ti_25_O_60_ is due to the eleven electrochemically active Mg ions per formula that allow multiple electron transfers in the demagnesiation process. The calculated capacity shows promising energy-storage capability, whereas many magnesium-based oxides reported in the literature exhibited poor performance.

**Fig. 7:**
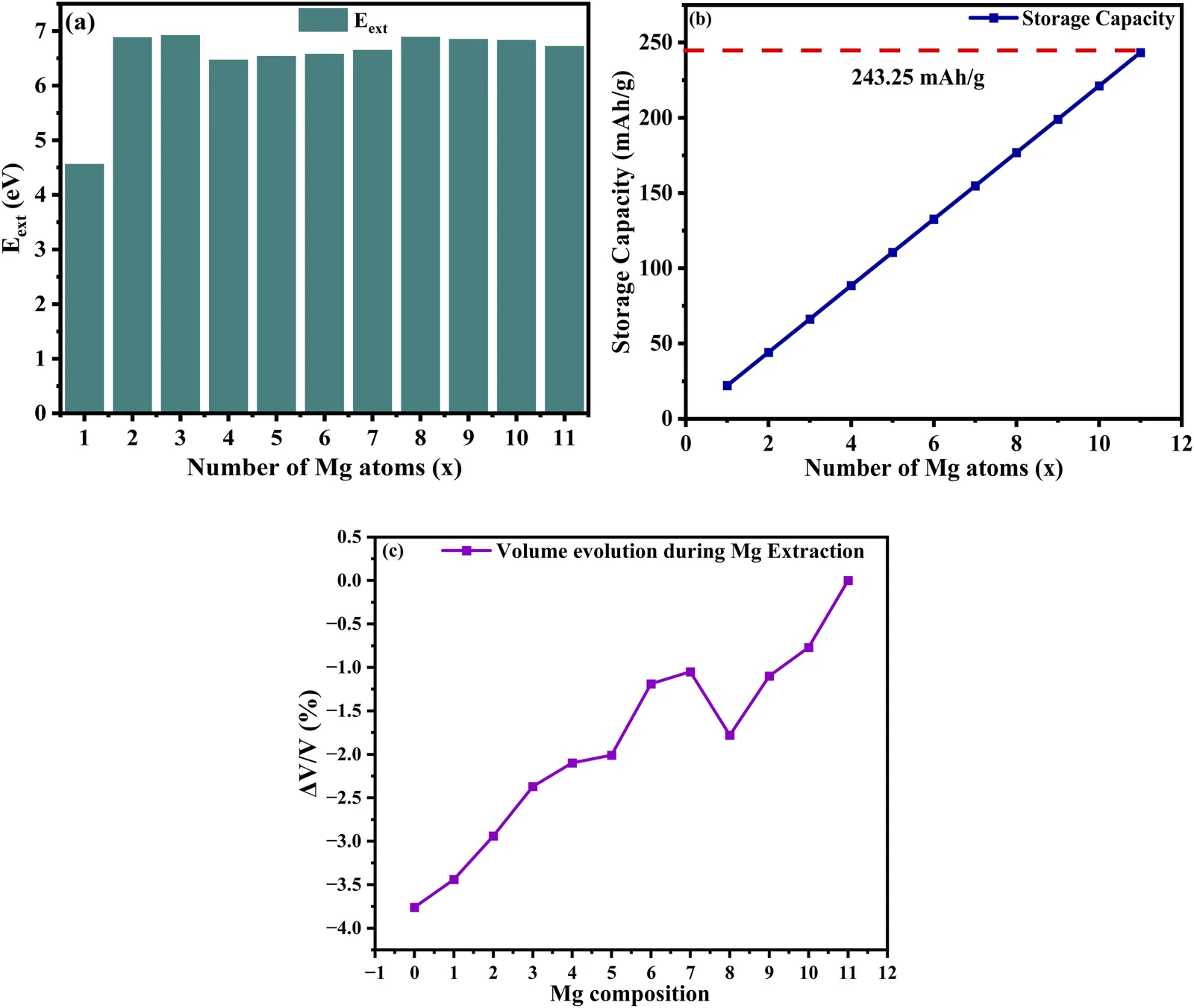
Calculated (a) *E*_ext_ (b) storage capacity and (c) volume change during Mg extraction from Mg_11_Ti_25_O_60_.

### 3.8 Open-circuit voltage (OCV) and site heterogeneity

OCV of the cathode material is an important factor that affects the operational voltage, energy density, and characteristics of rechargeable MIBs. It plays a crucial role in the energy storage performance and battery efficiency. The profile is a useful indicator of the thermodynamic stability of Mg insertion/extraction reactions, and this reflects the redox behavior of the host structure during electrochemical cycling.^55,122,123^ The voltage window calculated ranges from 2.33 V to 4.54 V, with an average operating voltage of 3.37 V, ideal for cathodes for MIBs, written in Table 1. While it undergoes a phase change give a long voltage plateau and has several peaks and valleys in its voltage profile during the demagnesiation process. It stems from the fact that Mg ions are present in non-equivalent sites in the crystal structure that contain sites of different bonding strengths. Thus, a different amount of energy is required for each Mg extraction operation, and the resulting calculated voltage profile will vary. Significant attraction between Mg ions and the Ti–O framework on specific lattice sites is reflected in the highest voltage of 4.54 V (*x* = 2) during the early step of Mg extraction. The voltage then drops and levels off between 3.2–3.5 V for the majority of the extraction process, implying an energetically favorable and reversible removal of Mg-ions. The other significant finding is that there is no sudden voltage drop upon demagnesiation, suggesting the structural stability of the Ti–O framework. This is an improvement over other reported cathodes for magnesium batteries, such as the Chevrel-phase cathodes, spinel oxides, and some vanadium-based materials. In addition, conventional spinel MgMn_2_O_4_ structures work only at low energies of 2.2–2.5 V, due to the limitations of strong lattice-ion trapping. Our elevated 3.37 V overcomes these traditional lower voltage limits, providing an important means of overcoming the slow transport and low energy density limitations of existing MIB systems.^16,107,108,124,125^ In contrast, the conventional cathodes encounter low energy and voltage capability.^6,63,126,127^

### 3.9 Volumetric and gravimetric energy density limits

Energy density is one of the most significant properties of a rechargeable battery cathode, which is the volume of energy stored per mass of the active material. The challenge lies in improving battery energy density to provide longer battery life, yet keeping battery weight and size low. Specific capacity and operating voltage are the main factors affecting the energy density of a cathode.^42,128^ The material Mg_11_Ti_25_O_60_ shows a very high theoretical energy density, about 819.75 W h kg^−1^, a specific capacity of 243.25 mA h g^−1^ and an average operating voltage of 3.37 V. The value is far larger than that of numerous cathodes recently reported for MIBs, thus demonstrating the promising energy-storage capability. This improved energy density is explained by the high operating voltage during demagnesiation, which allows to insert multiple Mg^2+^ inside the Ti–O framework. Moreover, electrochemical properties are desirable because of the combination of the diffusion characteristics, moderate migration barrier and stability of its electronic structure. As compared with conventional cathodes of magnesium-ion batteries, such as Chevrel-phase Mo_6_S_8_, spinel MgMn_2_O_4_ and a few compounds of vanadium, which usually store between 150 and 500 W h kg^−1^ of battery. Accordingly, it is concluded that Mg_11_Ti_25_O_60_ is a promising high-energy-density material for rechargeable MIBs.^129–131^

### 3.10 Solid-state magnesium diffusion kinetics

For MIBs, the ease with which the divalent magnesium ions move from one site to another directly influences the battery's power density and fast charging. To understand this atomic-scale movement inside the configuration, CI-NEB simulations were performed to find MEP. The calculations found a double-peak energy landscape rather than a single barrier, suggesting that the energy required for Mg^2+^ to pass through different types of coordination environments inside the titanium oxide network.^132–134^

The resultant migration path shows that the barrier for activation pathway is 0.62 eV. The electrostatic trapping that generally occurs in oxide and other host materials in the case of divalent Mg^2+^ is considerably reduced by the open 3D structure of Mg_11_Ti_25_O_60_. The calculated value of barrier 0.62 eV is found similar to the reported values on anatase TiO_2_ with value of 0.53 eV,^135^ and of VOPO_4_ exhibiting barrier of 0.69 eV.^136^ In case of V_2_O_5_ framework, the value of migration barrier has been reported as 1.26 eV which is higher than our findings.^137^ In contrast, the comparison with a closely related Mg_5_Ti_13_O_60_ system points to its smaller Mg^2+^ migration barrier of 0.23 eV.^26^ Although our calculated value of the present barrier is higher than that of Mg_5_Ti_13_O_60_, but is still similar to several reported Mg-ion cathode materials. The comparative summary on Mg^2+^ migration barrier is provided in Table 2. It is there concluded that, Mg^2+^ transport in Mg_11_Ti_25_O_60_ is kinetically feasible offering computed parameters comparable to the contemporary cathode materials (Fig. 8).

**Table 2:** Comparison of Mg_11_Ti_25_O_60_ with different cathode materials

Material	Capacity (mA h g^−1^)	Migration barrier (eV)	Diffusion coefficient (cm^2^ s^−1^)	Key finding	Reference
Mg_11_Ti_25_O_60_	243.25	0.62	1.16 × 10^−9^	High theoretical capacity with a stable Ti–O framework	Present work
Mg_5_Ti_13_O_30_	∼241	∼0.23	1.37 × 10^−9^	Stable Mg extraction and metallic conductivity	26
MgFeSiO_4_	∼30	<1	10^−13^	High theoretical capacity	145 and 159
Mg_2_Mo_6_S_8_ (Chevrel)	∼128	∼0.30	∼10^−11^ to 10^−13^	Benchmark Mg cathode with excellent reversibility	64
Interlayer-expanded MoS_2_	∼170, ∼223	∼0.25	∼10^−11^	Layered sulfide with favorable diffusion channels	160
Mg_2_Mo_3_O_8_	≈223	1.2 eV (Hop-1), 2.0 eV (Hop-2)	—	O–Mg–O dumbbell intermediate leads to very high Mg migration barriers	161
TiS_2_	∼120–150	∼0.45	10^−15^ to 10^−19^	Multi-phase structural evolution	22
MgMn_2_O_4_	∼150–250	0.38	—	3D hollow MgMn_2_O_4_ improves Mg^2+^ storage by shortening diffusion paths and enhancing capacity	110 and 162
Prussian blue and analogs	∼60–100	∼0.6	—	It has low intrinsic electronic conductivity	121

**Fig. 8:**
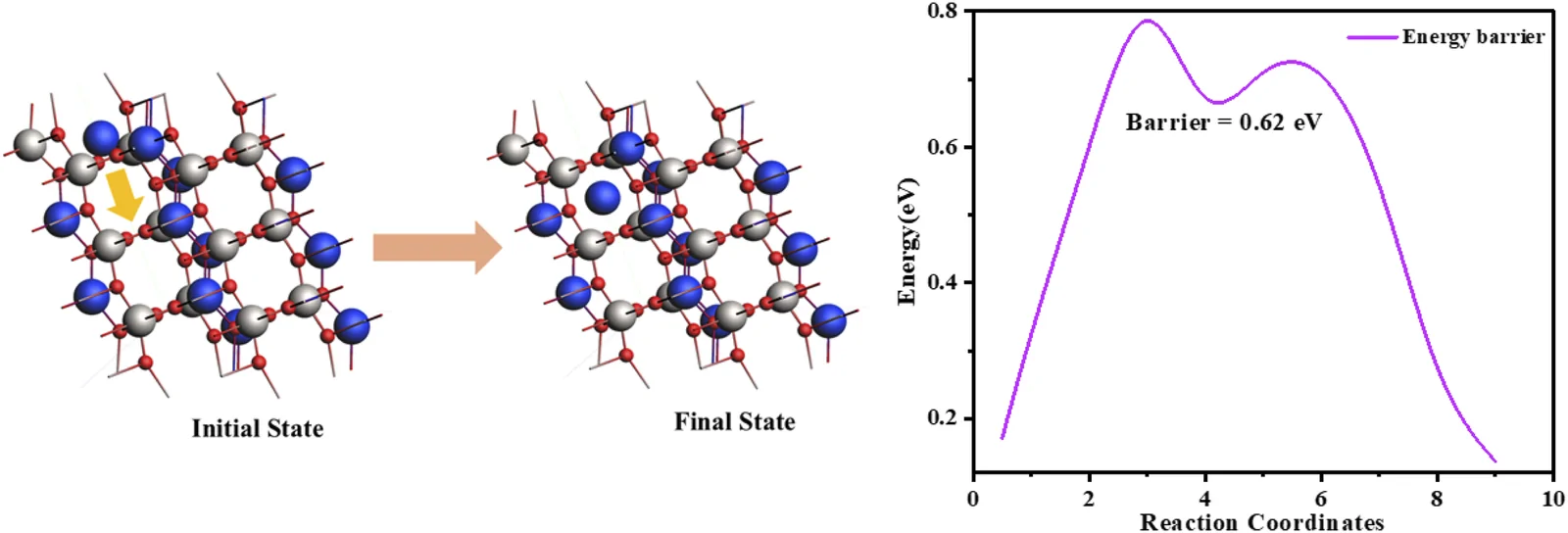
The calculated diffusion energy barrier for Mg_11_Ti_25_O_60_.

### 3.11 Diffusion coefficient and ionic conductivity analysis

The DC and *σ* are crucial factors for the transport of Mg^2+^ in the cathode components and regulate the electrochemical performance. The challenge for the fast conduction of Mg^2+^ is still very significant due to its high charge density and sluggish movement because it has a strong electrostatic attraction towards the surrounding O atoms of the host framework. In the conventional transition metal oxides, the strong attraction will hold the ions in place and reduce the diffusion coefficient to a very low range of 10^−12^–10^−15^ cm^2^ s^−1^.^138–142^

In this study, the calculated value of DC for Mg_11_Ti_25_O_60_ was found to be 1.16 × 10^−13^ m^2^ s^−1^. The MSD-based diffusion kinetics were calculated over the timeframe of the finite AIMD simulation at 300 K, and the primary rate-limiting migration pathway is captured by a CI-NEB barrier of 0.62 eV. This excellent value of DC exceeds those of the conventional cathode oxide materials which proves that this structure of Ti–O has large open structural channels. These most favorable pathways enable Mg^2+^ to glide in as smoothly as possible through the lattice without being strongly trapped electrostatically or experiencing kinetic blocking. It is also calculated that *σ* of the solution would be a very exceptional value of 2.82 × 10^−4^ S cm^−1^. On a comparative basis, most reported oxide cathodes have poor room values of *σ*, ranging from 10^−10^ to 10^−12^ S cm^−1^, and thus suffer from limited performance at high current rates. For example, glass ceramics of the composition Na_2_S–V_2_O_5_–P_2_O_5_ possess DC around 10^−12^ m^2^ s^−1^, which means the relatively slow diffusion of ions. For comparison, MgFeSiO_4_ is reported to have a DC of 10^−13^ m^2^ s^−1^. The strong interactions within the host structures result in a DC of the cathodes with MnO_2_ ranging between 10^−12^ and 10^−13^ cm^2^ s^−1^. In the same way, cathodes based on V_2_O_5_ have reported DC of 10^−13^ cm^2^ s^−1^. The layered TiS_2_ has an open crystal structure that results in DC of 10^−12^ cm^2^ s^−1^.^143–148^ Under these circumstances, the DC and outstanding *σ* make this combination desirable for practical applications in batteries.

The high performance of closely related compounds such as Mg_5_Ti_13_O_30_ validates the intrinsic potential of the magnesium titanate oxide family.^26^ It demonstrates that the stoichiometric and structural tuning within this class of oxide can further unlock superior electrochemical metrics for future battery applications. The Mg_11_Ti_25_O_60_ framework in the present work shows several distinct features, such as a large specific capacity of 243.25 mA h g^−1^ an average open-circuit voltage of 3.37 V, a relatively low Mg-ion migration barrier of 0.62 eV, metallic electronic behavior, and good thermal stability, and a low volume change during the extraction of Mg-ions. The results prove that Mg_11_Ti_25_O_60_ is a highly promising oxide cathode for Mg ion batteries and reinforce the family of high performance materials for Mg storage.

### 3.12 Quantum transport and cathode–electrolyte interphase (CEI) dynamics

The performance and long-term stability of rechargeable MIBs rely on the electrochemical performance and long-term stability of the cathode–electrolyte interphase (CEI) of the battery. A moderate thickness of CEI encourages efficient transportation of the Mg^2+^, prevents detrimental side reactions, and allows for structural integrity with repeated charge/discharge cycles. Moreover, the charge transport behavior at the interface of the cathode/electrolyte has essential role in the performance of the batteries. The electronic transport properties of the Mg_11_Ti_25_O_60_/ethylene carbonate system have been studied with quantum transport calculations of NEGF formalism.^110,149–152^

For high-voltage MIBs, the formation of CEI is a critical step, and the formation of cyclic carbonate electrolytes, such as Ethylene Carbonate (EC), is commonly found to undergo highly localized thermodynamic oxidative decomposition under severe potential profiles. The chemical interaction of these interacting components is important to study for the novel material proposal.^153–155^ Individual molecules of EC undergo a sequence of electron loss reactions that open the ring, when facilitated by the active surface centers of the host matrix, whose structure is highly active and under coordinated. This particular decomposition pathway breaks with the organic cyclic chains to give localized volatile components and the polymeric carbonate structures that immediately interact with the surface Mg and Ti boundaries. The resulting self-limiting interfacial layer is characterized by a protective CEI dominated by stable Mg–O coordination networks with other structural domains. CEI thickness and chemical resistance are critical for the cell performance, as the material becomes very thick or chemically resistant fast traps the charge carriers inside, leaving most of the continuous transport restricted to pass through the electrically conducting active material.^156^

The *I*–*V* steady-state characteristics are observed for the pure framework as shown in Fig. 9. A region with no redistributed current (the flat red line) is produced, showing a localized current suppression throughout the base voltage range, indicating a very symmetrical and stable electronic distribution under pristine equilibrium conditions. However, with the EC electrolyte, blue curves show a clear transformation as the interface turns into a very dynamic charge-transfer region. The electronic tunneling current doubles in symmetry, the current increases proportionally under forward bias and reverse bias, which is direct evidence of the accelerated injection of carriers and the initiation of electrochemical activity that occurs in concert with the solvation molecules.

**Fig. 9:**
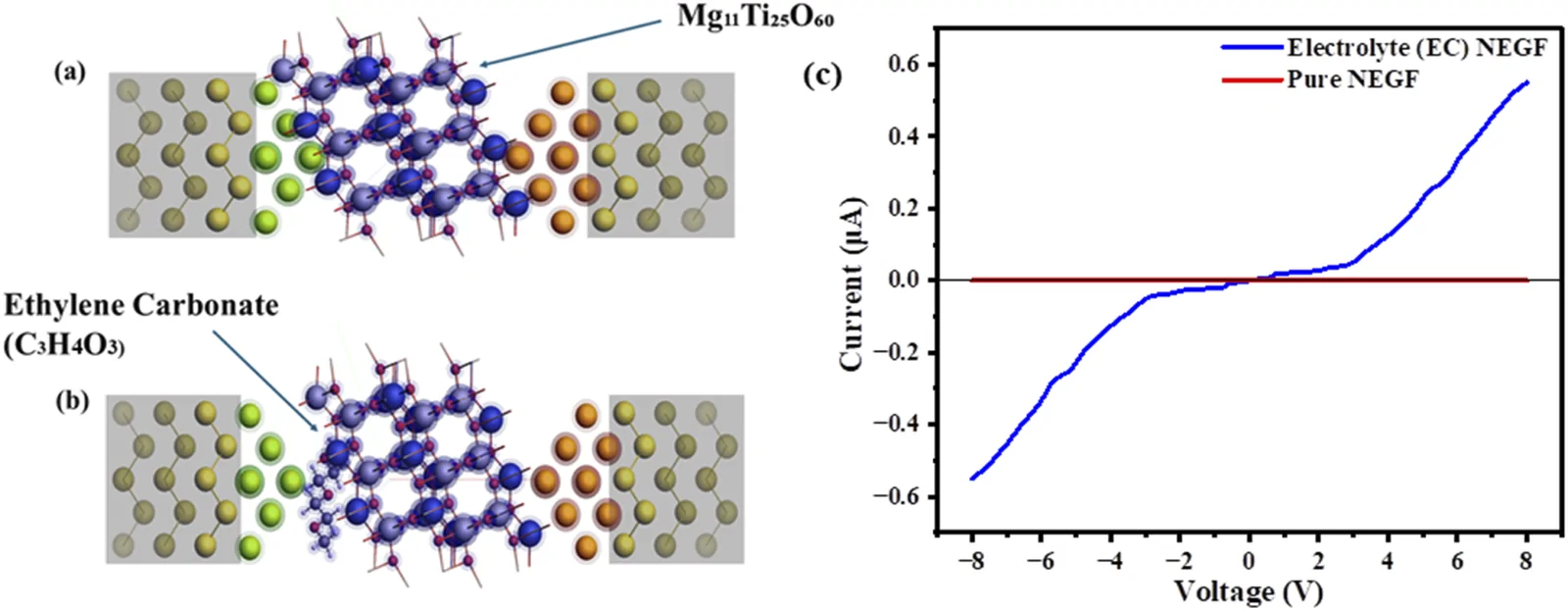
Schematic view of NEGF setup (a) Mg_11_Ti_25_O_60_ and (b) EC electrolyte with host placed between gold leads (c) *I*–*V* characteristics.

Several factors, such as the nature of interface, electrolyte and the operating voltage, are important parameters that govern the electrochemical behavior of the cathodes in magnesium ion batteries. For instance, Mo_6_S_8_ cathode is based on Grignard and chloride-based electrolyte, but other electrolytes often suffer from poor oxidative stability and passivate the Mg anode, leading to poor cycling performance. Likewise, for the spinel cathode MgMn_2_O_4_ in diglyme- or THF-based electrolyte, capacity degradation occurs because of the dissolution of manganese and the lack of protection of the interface. For V_2_O_5_ xerogels, however, operation under elevated voltage can increase this decomposition of the electrolyte, which will lead to an overgrown CEI and cause structural degradation during operation. On the other hand, the system studied in this paper shows advantageous interfacial properties characterized by improved charge transport properties. This favorable and stable behavior of the cathode towards the EC molecule should promote the formation of controlled CEI, which will favor the transport of Mg^2+^ and inhibit undesirable side reactions, hence ensuring a better electrochemical stability.^156–158^

To highlight the electrochemical performance of the engineered Mg_11_Ti_25_O_60_ host, its performance is compared to state-of-the-art MIBs cathodes in Table 2. All of the assembled benchmarks clearly state that the proposed scheme also overcomes the voltage limits and energy density restrictions of the standard multivalent intercalation matrices.

## 4. Conclusions

The study concludes that the proposed Mg_11_Ti_25_O_60_ displays promising characteristics for the development of next-generation rechargeable MIBs, making it a promising material for this purpose. The investigation confirms that the lattice framework of structurally robust titanium oxide can easily accommodate the insertion stress of divalent ions in keeping the structural integrity and volume stability during the sequential demagnesiation process. Strong local coulombic repulsion leads to distinct thermodynamic changes at certain intermediate insertion states, but the host framework remains very stable electrochemically without phase segregation or collapse. It satisfies the performance demands, overcoming the voltage limitations of traditional multivalent cathodes. It has an unusually high average open-circuit voltage of 3.37 V and a very high theoretical gravimetric energy density of 819.75 W h kg^−1^ and its theoretical capacity of 243.25 mA h g^−1^ remains stable. In addition to this, the framework demonstrates promising ionic conductivity of 2.82 × 10^−4^ S cm^−1^ and shows favorable electronic transport characteristics toward the electrolyte EC electrolyte interface. Critically, phonon dispersion analysis and molecular trajectories at 300 K are found to be thermally and dynamically stable, showing no imaginary frequency modes or framework degradation, in continuous grid conditions. Finally, these results provide a rigorous first-principles-based validation of magnesium titanate frameworks, laying down an explicit theoretical route to achieving high-energy-density, safe, and inexpensive solid-state multivalent energy storage devices.

## Conflicts of interest

The authors declare that they have no known competing financial interests or personal relationships that could have appeared to influence the work reported in this paper.

## Supplementary Material

RA-016-D6RA06938J-s001

## Data Availability

The data (generated *via* density functional based tight-binding (DFTB) module of the Amsterdam Modeling Suite (AMS/ADF) software package) supporting the findings of this study are available within the article. The raw computational datasets, optimized crystallographic structures, and input decks generated during the current investigation are available from the corresponding author upon reasonable request.
